# High-yielding continuous-flow synthesis of antimalarial drug hydroxychloroquine

**DOI:** 10.3762/bjoc.14.45

**Published:** 2018-03-08

**Authors:** Eric Yu, Hari P R Mangunuru, Nakul S Telang, Caleb J Kong, Jenson Verghese, Stanley E Gilliland III, Saeed Ahmad, Raymond N Dominey, B Frank Gupton

**Affiliations:** 1 Department of Chemistry and Department of Chemical and Life Science Engineering, Virginia Commonwealth University, 601 W. Main St., Richmond, VA 23220, USA

**Keywords:** antimalarial, API manufacturing, flow chemistry, hydrogenation, hydroiodic acid, hydroxychloroquine

## Abstract

Numerous synthetic methods for the continuous preparation of fine chemicals and active pharmaceutical ingredients (API’s) have been reported in recent years resulting in a dramatic improvement in process efficiencies. Herein we report a highly efficient continuous synthesis of the antimalarial drug hydroxychloroquine (HCQ). Key improvements in the new process include the elimination of protecting groups with an overall yield improvement of 52% over the current commercial process. The continuous process employs a combination of packed bed reactors with continuous stirred tank reactors for the direct conversion of the starting materials to the product. This high-yielding, multigram-scale continuous synthesis provides an opportunity to achieve increase global access to hydroxychloroquine for treatment of malaria.

## Introduction

**Figure 1:**
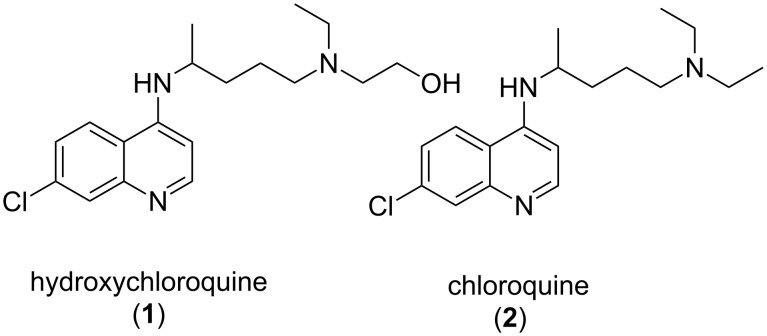
Commercially available antimalarial drugs.

Our research group has been focused on the development of new synthetic methods for the preparation of a variety of active pharmaceutical ingredients for global health applications by employing the principles of process intensification [1–3]. In 2016, estimated 212 million cases of malaria, including 429,000 fatalities, were reported worldwide, with the majority of these cases occurring in sub-Saharan Africa and Southern Asia [4]. The malaria epidemic is particularly difficult to control due to the multidrug resistant nature of the malaria parasite *Plasmodium falciparum*. Hydroxychloroquine (**1**) is an antimalarial drug developed for both treatment and prevention of the disease in response to the widespread malaria resistance to chloroquine (**2**, Figure 1) [5–6].

Additionally, hydroxychloroquine (**1**, HCQ) is an effective non-steroidal drug with anti-inflammatory activity for the treatment of rheumatoid arthritis (RA) in patients with cardiovascular disease [7–9]. The World Health Organization has identified HCQ (**1**) as an essential antimalarial medication for a basic healthcare system, but global access to HCQ (**1**) has been hindered by high manufacturing costs of the API. Thus, the development of cost effective synthetic strategies to increase the global access to this important global health drug is of great importance. Effective strategies for accomplishing such objectives often include identifying more cost effective starting materials and reagents, simplifying the synthetic route in terms of reducing the total number of steps as well as reducing the cost and improving the efficiency of individual steps.

Flow chemistry methodologies have been increasingly investigated in recent years in the pharmaceutical industry for multistep preparations of highly-complex natural products and APIs [10–19]. Advantages include precise control of key reaction parameters such as heat and pressure, improved heat and mass transfer capabilities for better thermal control, enhanced selection of kinetically controlled products to potentially maximize conversion, smaller equipment footprint, and increased safety profiles when working with hazardous materials and reaction conditions [20]. These advantages often result in flow processes being significantly more efficient, as well as less costly, when compared to batch processes.

**Scheme 1:**
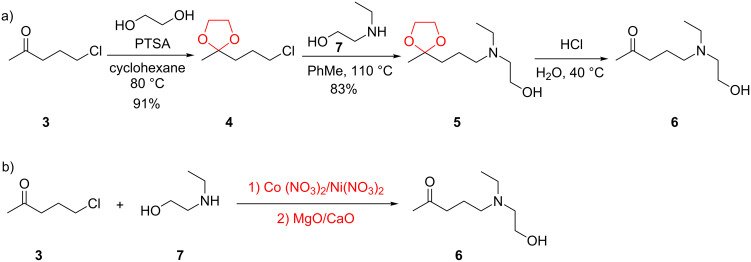
Current batch syntheses of the key intermediate 5-(ethyl(2-hydroxyethyl)amino)pentan-2-one (**6**).

HCQ (**1**) is currently produced via the batch method shown in Scheme 1. Therefore, continuous-flow chemistry approaches to synthesizing HCQ (**1**) offer a great potential to maximize the efficiency, and thus significantly reduces the overall manufacturing costs of this important medicine.

**Scheme 2:**
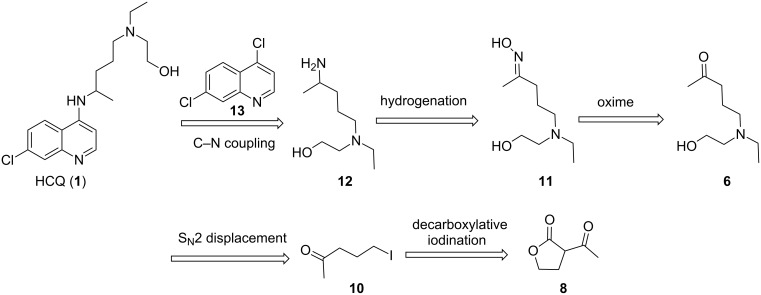
Retrosynthetic strategy to hydroxychloroquine (**1**).

The commercial HCQ synthesis employs a key intermediate, 5-(ethyl(2-hydroxyethyl)amino)pentan-2-one (**6**), which is a major cost driver in the process. The protection–deprotection strategy of chloro-ketone starting material **3** used in the commercial route (Scheme 1a) [21] has been targeted as a significant opportunity for optimization. While the recent improved route (Scheme 1b) by Li and co-workers [21] eliminates the protection–deprotection steps, its use of a complex multi-transition-metal-catalyst system to achieve direct S_N_2 substitution of the chlorine on **3** by amine **7**, is sub-optimal [22–23]. With these issues in mind, we carried out a retrosynthetic analysis (Scheme 2) in which **10**, an iodo analogue to the starting material **3**, could be generated in a single step via a decarboxylative ring-opening of α-acetyl butyrolactone **8**. The iodo analogue **10** could then be used without isolation to prepare compound **6**.

It is well known that the direct one-step reductive amination of **6** to give **12** can be accomplished by simple heterogeneous reduction with H_2_/Raney-nickel [24]. However, THF is employed in all of our prior flow steps and is a poor choice as a solvent for the reductive amination step due to limited solubility of ammonia in THF. H_2_/Raney-nickel reductions are often carried out in alcoholic media where much higher concentrations of ammonia are achievable but would require a solvent exchange. There are many reports of continuous-flow chemistry methods for reductive amination of ketones [25–31]; however, such processes typically require soluble reductants such as diisobutylaluminium hydride (DIBAL-H), superhydrides, or supported borohydride species [32–36]. Although these approaches are effective, they are significantly more costly than using simple heterogeneous reduction with H_2_/Raney-nickel. Therefore, we explored an alternate strategy: simple conversion of the ketone group of **6** to oxime **11,** followed by reduction to give 5-(ethyl(2-hydroxyethyl)amino)-2-aminopentane (**12**). We have found H_2_/Raney-nickel efficiently reduces **11** to **12** with THF as the solvent in a continuous stirred tank reactor (CSTR).

The last step requires the reaction of **12** with 4,7-dichloroquinoline (**13**) which when used neat takes 24–48 hours at 120–140 °C to give 75–80% yield of HCQ (**1**) [37]. We have found that this step can be accelerated by employing K_2_CO_3_/triethylamine, to facilitate the formation of **1**, resulting in a comparable yield in less than 6 hours. Thus, we have integrated the continuous preparation of reaction with our new efficient continuous-flow synthesis of **12** with the final step by using a CSTR to accommodate the longer reaction time required to produce HCQ (**1**).

## Results and Discussion

Initial optimization efforts to prepare **6** (Scheme 1) revealed poor reactivity of starting material **3**, so we pursued the iodo analogue of **3**, 5-iodopentan-2-one (**10**) as an alternative. By optimizing the reaction concentration, we have also shown that (see Table S1 in Supporting Information File 1) **10** reacts rapidly and cleanly with **7** under flow conditions to give **6** in high yield (>80%). Furthermore, we have developed and optimized a continuous synthesis of **10** (Table 1), wherein hydroiodic acid is reacted with neat 3-acetyldihydrofuran-2(3*H*)-one (**8**) to provide a rapid route to **10** which is significantly higher in yield than in previously reported syntheses [38–39]. Initial results using diluted hydroiodic acid (20–40%) provided only modest conversion to product over a range of temperatures (Table 1, entries 1–5); however, the use of 55% hydroiodic acid (Table 1, entries 6–8) was found to give near quantitative conversion. The reaction profile was monitored using GC–MS and ^1^H NMR – no intermediates were observed under these conditions. Optimization of the flow rate with 55% hydroiodic acid (Table 1, entries 6–8) revealed that a flow rate of 1.0 mL min^−1^ (*t_R_* = 5 min) gave an isolated yield of 89%.

**Table 1:** Optimization of the flow process for the synthesis of **10**.

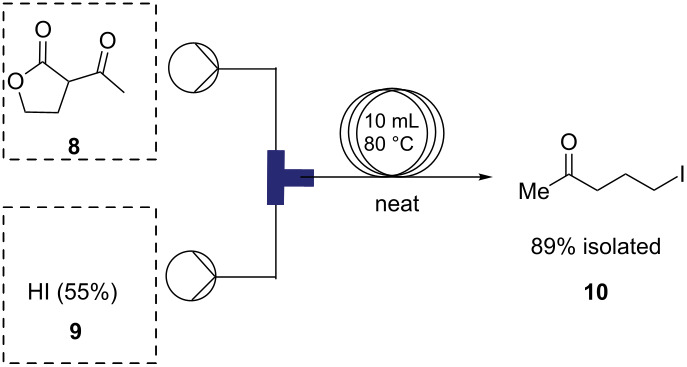					

entry	HI [aqueous %]	temp (°C)	*t*_R_ = min	pressure (bar)	conv.^a^ (%)

1	20	r.t.	5	1.5	5
2	20	40	5	2.0	31
3	20	80	5	2.0	34
4	40	80	5	2.0	43
5	40	80	5	2.5	46
6	55	80	5	3.0	98 (89%)^b^
7	55	80	2.5	3.0	91
8	55	80	10	3.0	92

^a^Conversion determined by GC–MS and ^1^H NMR. ^b^Isolated yield.

**Scheme 3:**
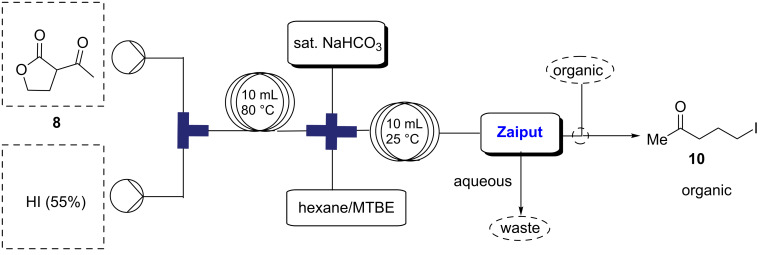
Schematic representation for continuous in-line extraction of **10**.

Due to the need to use an excess of hydroiodic acid it is important to remove its excess from the eluting reaction stream before telescoping into the next step in flow. The product stream containing crude **10** was mixed in-line with methyl *tert*-butyl ether (MTBE) and saturated NaHCO_3_ before phase separation using a hydrophobic, membrane-based separator (Zaiput) [40] (Scheme 3) to afford purified **10** in the organic phase. A loss of 5–10% of product to the water layer was observed, however, this was deemed adequate as it prevented the need for a complete work-up step in batch.

In the next step **6** was reacted with hydroxylamine, which was facilitated by passing through a packed-bed of K_2_CO_3_ to give oxime **11** (Table 2). As was seen with the reaction to produce **6** (Table S1 in Supporting Information File 1), reactant concentrations also had a dramatic effect on the oxime formation. A series of experiments were conducted to optimize the continuous formation of **11**. Reaction yields were modest at lower reactant concentrations across several temperatures and residence times (Table 2). Conversion to **11** increased when reactant concentrations were increased (9% at 0.1 M to 72% at 1 M, Table 2, entries 1–6). Optimization of the flow rate with 1 M concentrations of each reactant (Table 2, entries 6–8) showed that a flow rate of 1.0 mL min^−1^ (*t_R_* = 20 min) was optimal, giving an isolated yield of 78% (Table 2, entry 7).

**Table 2:** Schematic representation for the continuous telescoped process to synthesize **11**.

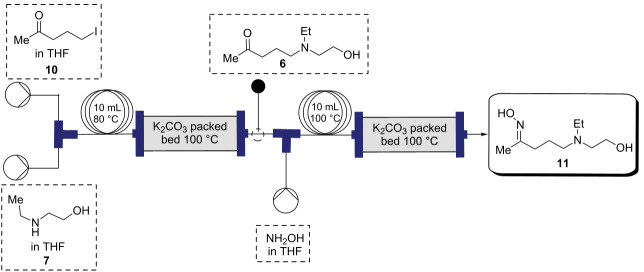				

entry	concentration^a^	temp (°C)	*t*_R_ = min	conv. of **11** (%)^b^

1	0.1 M	100	10	9
2	0.2 M	100	10	16
3	0.4 M	100	10	34
4	0.6 M	100	10	37
5	0.8 M	100	10	62
6	1.0 M	100	10	72
7	1.0 M	100	20	85 (78)^c^
8	1.0 M	100	40	76

^a^Concentration of **10** , **7** and hydroxylamine. ^b^Conversion determined by GC–MS and ^1^H NMR. ^c^Isolated yield.

The reductive amination of **11** performed in the first generation batch process was carried out using Raney-nickel at 80 °C and 10 bar hydrogen pressure for 4–6 h [21–24]. In order to perform this step in a continuous fashion, a continuous stirred tank reactor [25,41] was employed (Table 3). Materials were delivered to the CSTR vessel through an HPLC pump and were reacted under hydrogen pressure with mechanical stirring. The dip tube in the CSTR was outfitted with a fritted metal filter, allowing for retention of the heterogeneous catalyst within the CSTR vessel. Optimization of this CSTR-based flow process (Table 3) showed near quantitative yields of **12** over a broad range of oxime **11** reactant concentrations. An optimum residence time was determined to be 4 hours.

**Table 3:** Optimization of the flow process for the reductive amination of **12** using a CSTR.

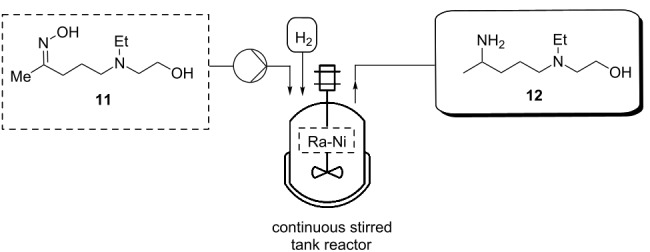					

entry	oxime [concentration]	temp. (°C)	pressure (bar)	*t*_R_ = hours	conv. of **12** (%)^a^

1	0.05 M	80	10	4	94%
2	0.25 M	80	10	4	96%
3	0.5 M	80	10	4	97%
4	2.0 M	80	10	4	98% (89%)^b^
5	2.0 M	80	10	2	56%
6	2.0 M	80	10	1	46%

^a^Conversion determined by GC–MS and ^1^H NMR. ^b^Isolated yield.

**Scheme 4:**
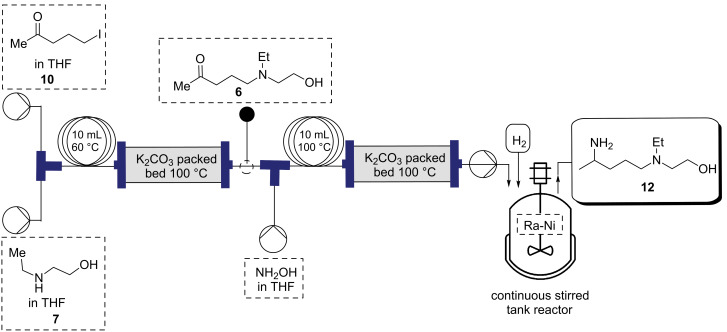
Optimization of the flow process for the synthesis of **12**.

After optimizing the individual steps up to compound **12** the entire reaction was telescoped into a continuous reaction process that convert **10** and **6** into **12** (Scheme 4) with an overall isolated yield of 68% for compound **12**.

With an optimized continuous process for producing the key intermediate **12** in-hand the reaction conditions for the conversion of **12** to HCQ (**1**) were examined. In the commercial process this step is carried out in batch under neat reactant conditions and requires a relatively long reaction time of 24–48 h [42–44]. In order to convert this step to a flow chemistry method, we selected to employ a CSTR (Table 4). This final step, transforming **13** and **12** into **1**, was first investigated in batch to optimize the conditions before implemented in a CSTR.

**Table 4:** Optimization of the reaction conditions for the preparation of hydroxychloroquine (**1**).^a^

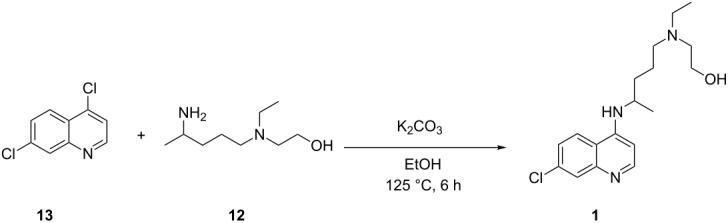				

entry	base	solvent	temp. (°C)	conv. to **1** (%)^b^

1	NaOH	EtOH	125	30
2	KOH	EtOH	125	35
3	K_2_CO_3_	EtOH	125	82
4	Et_3_N	EtOH	125	61
5	DIPEA	EtOH	125	55
6	K_2_CO_3_/Et_3_N	EtOH	125	88 (78)^c^

^a^Reaction conditions: 4,7-dichloroquinoline (**13**, 1.0 equiv), base (1.0 equiv), amine **12** (1.2 equiv). ^b^Conversion determined by HPLC and ^1^H NMR, ^c^Isolated yield.

Process optimization for the final step started with the screening of the effect of solvents and base(s) on the yield of HCQ (**1**). Screening of different polar–protic and non-protic solvents (see Table S2 in Supporting Information File 1) demonstrated that ethanol is the most effective for this transformation. During the screening of bases, the p*K*_a_ of the amine and alcohol groups present in compound **12** were given careful consideration in order to minimize C–O bond formation (Table 4). NaOH or KOH in ethanol gave low (<40%) conversion, whereas using K_2_CO_3_ in ethanol gave 82% conversion to HCQ (Table 4, entry 3). Attempts with organic bases (Table 4, entries 5 and 6) resulted in only moderate conversions to the desired product; however, using a 1:1 mixture of K_2_CO_3_/Et_3_N (1:1) resulted in 88% conversion (Table 4, entry 6) to **1**, with corresponds to an isolated yield of 78%.

## Conclusion

In summary, we have developed a high-yielding continuous-flow process for the synthesis of hydroxychloroquine (**1**, HCQ) by optimizing continuous-flow methods for the synthesis of key intermediates **6** and **12**. Additionally, we have developed and optimized flow-chemistry conditions for performing reductive amination of **11** using Raney-nickel as catalyst in a continuous stirring tank reactor (CSTR) for the synthesis of compound **12**, and have incorporated it into a fully continuous telescoped process for the synthesis of **12** from lactone **8** and aminoethanol **7**. Feeding the output stream containing **12** from the above CSTR into a second CSTR in which **12** is converted to HCQ (**1**) provides a completely continuous-flow process for producing HCQ (**1**) from readily available starting materials. This efficient process has the potential to increase the global access to this strategically important antimalarial drug. We are currently working to demonstrate that this fully integrated continuous-flow process for the synthesis of HCQ (**1**) can be scaled to commercial operations.

## Experimental

### General information

All reactions for the preparation of substrates were performed in standard, dry glassware under an inert atmosphere of nitrogen or argon unless otherwise described. All starting materials and reagents were purchased from commercial sources and were used as received unless otherwise noted. ^1^H and ^13^C NMR spectra were recorded using 600 MHz spectrometers. Chemical shift (δ) values are given in ppm, and coupling constants (*J*) are given in Hz. The 7.26 ppm resonance of residual CHCl_3_ (or 0 ppm of TMS) for proton spectra and the 77.23 ppm resonance of CDCl_3_ for carbon spectra were used as internal references. Continuous-flow experiments were carried out using the E-series flow reactor instrument purchased from Vapourtec Ltd. PFA tubing (1/16 OD × 1 mm ID) was used for all reactor coils in flow experiments. Most of the reagents and starting materials were purchased from commercial sources and used as received. All HPLC chromatograms were recorded on an Agilent Technologies 1260 Infinity instrument with a Poroshell 120 EC-C18 column (4.6 × 50 mm, 2.7 micron). Continuous flow hydrogenation was performed using a FlowCAT instrument.

#### Synthesis of 5-iodopentan-2-one (**10**)


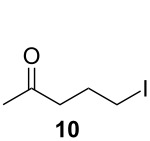


Two solutions, 2-acetylbutyrolactone (**8**, 1.176 mL, 10.35 mmol, 1.0 equiv) and hydroiodic acid (55% aqueous solution) were pumped at 1.0 mL min^−1^ using peristaltic pumps through a 10 mL coil (residence time, *t*_R_ = 5 min) at 80 °C. The completion of the reaction was monitored using GC–MS. Complete consumption of starting material was observed. The reaction mixture was cooled to room temperature and sodium hydrogencarbonate was added until neutralized at pH 7. The crude mixture was extracted with hexanes/MTBE and the combined organic phase was dried over anhydrous sodium sulfate and evaporated in vacuo to dryness yielding the desired product as a light brown liquid (14.72 g, 89%). ^1^H NMR (600 MHz, CDCl_3_) δ 3.22 (t, *J* = 6.9 Hz, 2H), 2.59 (t, *J* = 6.9 Hz, 2H), 2.17 (s, 3H), 2.06 (quin, *J* = 7.0 Hz, 2H); ^13^C NMR (125 MHz, CDCl_3_) δ 207.4, 44.0, 30.3, 27.2, 6.7. Spectra were obtained in accordance with those previously reported [3].

#### Synthesis of 5-(ethyl(2-hydroxyethyl)amino)pentan-2-one (**6**)


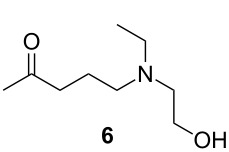


**Telescope of compound 6:** Prior to the start of the experiment, the flow reactor unit was rinsed with dry THF and flushed with nitrogen gas. At room temperature, the stock solutions of 5-iodopentan-2-one (**10**, 1.0 M) and 2-(ethylamino)ethan-1-ol (**7**) in THF solution (1.0 M) were streamed in at 0.5 mL min^−1^ via a T-piece into a 10 mL reactor coil (*t*_R_ = 10 min) and passed through a packed bed reactor of potassium carbonate at 100 °C. The output solution was collected and quenched with a saturated solution of ammonium chloride. The aqueous phase was extracted by DCM (3 × 50 mL) and the organic layers were combined, dried over sodium sulfate, and evaporated in vacuo to give a light brown liquid (14.05 g, 86%). ^1^H NMR (600 MHz, CDCl_3_) δ 3.53 (t, *J* = 5.2 Hz, 2H), 2.58 (m, 3H), 2.53 (m, 2H), 2.45 (t, *J* = 6.7 Hz, 4H), 2.59 (t, *J* = 6.9 Hz, 2H), 2.17 (s, 3H), 2.07 (quin, *J* = 7.0 Hz, 2H); ^13^C NMR (125 MHz, CDCl_3_) δ 208.9, 58.6, 55.0, 52.4, 47.2, 41.3, 30.0, 21.2, 11.7. Spectra were obtained in accordance with those previously reported [38–39].

#### Synthesis of (*E*)-5-(ethyl(2-hydroxyethyl)amino)pentan-2-one oxime (**11**)


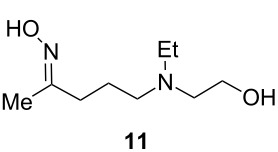


**Flow**: Prior to the start of the experiment, the flow reactor unit was rinsed with dry THF and flushed with nitrogen gas. At room temperature, the stock solutions of 5-iodopentan-2-one (**10**, 1.0 M) and 2-(ethylamino)ethan-1-ol (**7**) in THF solution (1.0 M) were streamed in at 0.5 mL min^−1^ via a T-piece into a 10 mL reactor coil (*t*_R_ =10 min) and passed through a packed bed reactor of potassium carbonate. The output solution was streamlined with hydroxylamine (1.0 M) at 1.0 mL min^−1^ via a T-piece into a 10 mL reactor coil (*t*_R_ =10 min) and passed through a packed bed reactor of potassium carbonate at 100 °C. The reaction mixture was then concentrated in vacuo, taken up in dichloromethane (3 × 20 mL) and concentrated under reduced pressure to yield **11** as light brown liquid. The crude product was used in the next step without further purification.

#### Synthesis of 2-((4-aminopentyl)(ethyl)amino)ethan-1-ol (**12**)


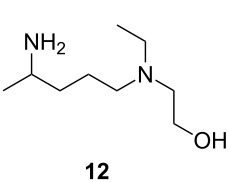


**Flow**: The synthesis of compound **12** was performed in a HEL continuous stirred tank reactor (CSTR) with a reaction volume of 150 mL. The reaction vessel was first charged with Raney-nickel (1.0 g). The Raney-nickel catalyst was retained in the CSTR by the 2 µm metal filter frit on the dip tube of the exit stream. The reaction mixture, consisting of compound **11** (0.05–2.0 M) in THF, was pumped by a HPLC pump set at a flow rate of 0.6–2.5 mL min^−1^ into the reaction vessel. The reaction pressure was set to 10 bar of hydrogen supplied by hydrogen gas (ultrahigh purity) at a flow rate of 0.5 mL min^−1^. The reaction temperature was set to 80 °C which was controlled by a thermocouple positioned in the reaction mixture. The reaction was stirred with mechanical stirring (750 rpm) to provide proper mixing. Two thermocouples were used to control the reaction volume in the reactor by setting a level control of −3 °C. The lower thermocouple constantly measured and controlled the reaction temperature and the upper thermocouple measured the temperature at approximately 150 mL reactor volume. When the two thermocouples were within 3 °C, the level control ‘opened’ the exit stream dip tube to allow products to exit the reactor, or ‘closed’ the exit stream dip tube to allow the reactor to fill when the temperature difference between the two thermocouples was greater than 3 °C. The product was collected after a full reaction volume of material (150 mL) had passed through the CSTR indicating that steady-state was reached. The reaction was monitored by liquid chromatography and ^1^H NMR. The reaction mixture was filtered through a celite pad and dried under reduced pressure. The solution was extracted with water (10 mL) and dichloromethane (3 × 20 mL). The organic layers were combined, washed with brine and dried over sodium sulfate and evaporated in vacuo. The resulting oil was fractionally distilled to give a colorless liquid (16.83 g, 84%). ^1^H NMR (600 MHz, CDCl_3_) δ 3.53 (t, *J* =5.3 Hz, 2H), 2.89 (sx, *J* = 6.4 Hz, 1H), 2.57 (t, *J* = 5.5 Hz, 2H), 2.55 (t, *J* = 7.0 Hz, 2H), 2.45 (t, *J* = 7.0 Hz, 2H), 1.55–1.44 (m, 2H), 1.36–1.27 (m, 2H), 1.22 (t, *J* = 7.1 Hz, 2H), 1.07 (d, *J* = 7.1 Hz, 2H), 1.00 (t, *J* = 7.1 Hz, 2H); ^13^C NMR (125 MHz, CDCl_3_) δ 58.2, 54.9, 53.2, 46.9, 46.7, 36.6, 23.8, 22.4, 10.6. Spectra were obtained in accordance with those previously reported [38–39].

#### Synthesis of 2-((4-((7-chloroquinolin-4-yl)amino)pentyl)(ethyl)amino)ethan-1-ol (**1**)


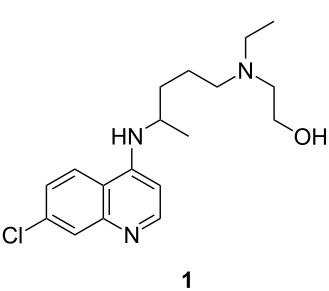


**Batch**: In a CSTR reactor, to a mixture of 4,7-dichloroquinoline (200 mg, 1.0 mmol), compound **12** (208 mg, 1.2 mmol), triethylamine (0.069 mL, 0.5 mmol, 0.5 equiv) and potassium carbonate (69 mg, 0.5 mmol, 0.5 equiv) was added ethanol (1.0 mL). The ethanol was distilled off from the reaction mixture and kept under nitrogen atmosphere (15 psi). The reaction was left at 125 °C in the nitrogen atmosphere for 6 h. After cooling, the mixture was transferred into a separatory funnel using 1 M aqueous sodium hydroxide (5 mL) and dichloromethane (2 × 20 mL). The organic phases were separated and the aqueous phase was re-extracted with dichloromethane (2 × 10 mL). The organic layers were combined and dried over sodium sulfate and evaporated in vacuo*.* The crude material was purified using flash chromatography with DCM/Et_3_N/MeOH 95:3:2 to give a white solid (0.263 g, 78%). ^1^H NMR (600 MHz, CDCl_3_) δ 8.48 (d, *J* = 5.4 Hz, 1H), 7.93 (d, *J* = 5.4 Hz, 1H), 7.70 (d, *J* = 9.2 Hz, 1H), 7.34 (dd, *J* = 8.8, 7.3 Hz, 1H), 6.39 (d, *J* = 5.4 Hz, 1H), 4.96 (d, *J* = 7.5 Hz, 1H), 3.70 (sx, *J* = 6.8 Hz, 1H), 3.55 (m, 2H), 2.57 (m, 5H), 2.49 (m, 2H), 1.74–1.62 (m, 1H), 1.65–1.53 (m, 3H), 1.31 (d, *J* = 6.9 Hz, 3H), 1.24 (d, *J* = 7.2 Hz, 2H); ^13^C NMR (125 MHz, CDCl_3_) δ 152.2, 149.5, 149.2, 135.0, 129.0, 125.4, 121.2, 117.4, 99.4, 58.6, 54.9, 53.18, 48.5, 47.9, 34.5, 24.1, 20.6, 11.9. Spectra were obtained in accordance with those previously reported [38–39].

## Supporting Information

File 1 Additional experimental descriptions and NMR spectra.

## References

[1] Verghese J, Kong C J, Rivalti D, Yu E C, Krack R, Alcázar J, Manley J B, McQuade D T, Ahmad S, Belecki K (2017). Green Chem.

[2] Kong C J, Fisher D, Desai B K, Yang Y, Ahmad S, Belecki K, Gupton B F (2017). Bioorg Med Chem.

[3] Martin A D, Siamaki A R, Belecki K, Gupton B F (2015). J Flow Chem.

[4] World Health Organization (2016). WHO Malaria Report 2016.

[5] Surrey A-R, Hammer H F (1950). J Am Chem Soc.

[6] Bailey D M (1969). J Med Chem.

[7] Pavelka K, Sen K P, Pelísková Z, Vácha J, Trnavský K (1989). Ann Rheum Dis.

[8] Hage M P, Al-Badri M R, Azar S T (2014). Ther Adv Endocrinol Metab.

[9] Poorvashree J, Suneela D (2017). Drug Delivery Transl Res.

[10] Belecki K, Gupton B F (2015). Continuous Processing in Drug Discovery. Green Chemistry Strategies for Drug Discovery.

[11] Baumann M, Baxendale I R (2015). Beilstein J Org Chem.

[12] Baxendale I R, Brocken L, Mallia C J (2013). Green Process Synth.

[13] Opalka S M, Park J K, Longstreet A R, McQuade D T (2013). Org Lett.

[14] Alonso N, Miller L Z, Muñoz J d M, Alcázar J, McQuade D T (2014). Adv Synth Catal.

[15] McQuade D T, Seeberger P H (2013). J Org Chem.

[16] Poechlauer P, Colberg J, Fisher E, Jansen M, Johnson M D, Koenig S G, Lawler M, Laporte T, Manley J, Martin B (2013). Org Process Res Dev.

[17] Poechlauer P, Manley J, Broxterman R, Gregertsen B, Ridemark M (2012). Org Process Res Dev.

[18] Britton J, Raston C L (2017). Chem Soc Rev.

[19] Plutschack M B, Pieber B, Gilmore K, Seeberger P H (2017). Chem Rev.

[20] Gutmann B, Cantillo D, Kappe C O (2015). Angew Chem, Int Ed.

[21] You H, Liu Y, Ning F, Zheng Z, Yu Q, Niu X, Li C (2015).

[22] Glenn J S, Pham E A (2017). Use of chloroquine and clemizole compounds for treatment of inflammatory and cancerous conditions PCT. Int. Appl..

[23] Min Y S, Cho H-S, Mo K W (2010). New preparation of hydroxychloroquine. PCT Int. Appl..

[24] Ashok K, Dharmendra S, Snajay N, Sanjay B, Atul J (2005). An improved process for the preparation of 7-chloro-4-(5-N-ethyl-N-2-hydroxyethylamine)-2-pentyl]aminoquinoline and its intermediates.

[25] Continuous flow chemistry catalytic reactions with FlowCAT.

[26] Jensen R K, Thykier N, Enevoldsen M V, Lindhardt A T (2017). Org Process Res Dev.

[27] Falus P, Boros Z, Hornyánszky G, Nagy J, Darvas F, Ürge L, Poppe L (2011). Tetrahedron Lett.

[28] Chi Y, Zhou Y-G, Zhang X (2003). J Org Chem.

[29] Hoffmann S, Seayad A M, List B (2005). Angew Chem, Int Ed.

[30] Kitamura M, Lee D, Hayashi S, Tanaka S, Yoshimura M (2002). J Org Chem.

[31] Kadyrov R, Riermeier T H (2003). Angew Chem, Int Ed.

[32] Abdel-Magid A F, Carson K G, Harris B D, Maryanoff C A, Shah R D (1996). J Org Chem.

[33] Gilmore K, Vukelić S, McQuade D T, Koksch B, Seeberger P H (2014). Org Process Res Dev.

[34] Webb D, Jamison T F (2012). Org Lett.

[35] Fan X, Sans V, Yaseneva P, Plaza D D, Williams J, Lapkin A (2012). Org Process Res Dev.

[36] Kirschning A, Monenschein H, Wittenberg R (2001). Angew Chem, Int Ed.

[37] Blaney P M, Byard S J, Carr G, Ellames G J, Herbert J M, Peace J E, Smith D I, Michne W F, Sanner M S (1994). Tetrahedron: Asymmetry.

[38] Cornish C A, Warren S (1985). J Chem Soc, Perkin Trans 1.

[39] Münstedt R, Wannagat U, Wrobel D (1984). J Organomet Chem.

[40] Hamlin T A, Lazarus G M L, Kelly C B, Leadbeater N E (2014). Org Process Res Dev.

[41] Chapman M R, Kwan M H T, King G, Jolley K E, Hussain M, Hussain S, Salama I E, Niño C G, Thompson L A, Bayana M E (2017). Org Process Res Dev.

[42] Xiao H, Tong R, Liao Z, Chuan J, Zhang L, Zhang Y, Bian Y (2014). Hydroxychloroquine linolenate and synthesis method thereof.

[43] Sleightholm R, Yang B, Yu F, Xie Y, Oupický D (2017). Biomacromolecules.

[44] Ansari A M, Craig J C (1994). J Chem Soc, Perkin Trans 2.

