# Supplementary material for: High-yielding continuous-flow synthesis of antimalarial drug hydroxychloroquine
Source: Beilstein J Org Chem. 2018 Mar 8;14:583–92. doi: 10.3762/bjoc.14.45 (PMC5852550; doi:10.3762/bjoc.14.45)
Supplement: File 1 — Additional experimental descriptions and NMR spectra. [file Beilstein_J_Org_Chem-14-583-s001.pdf]

Supporting Information

for

High-yielding continuous-flow synthesis of antimalarial drug hydroxychloroquine

Eric Yu‡, Hari P. R. Mangunuru‡, Nakul S. Telang, Caleb J. Kong, Jenson Verghese, Stanley E. Gilliland III, Saeed Ahmad, Raymond N. Dominey and B. Frank Gupton*

Address: Department of Chemistry and Department of Chemical and Life Science Engineering, Virginia Commonwealth University, 601 W. Main St., Richmond, VA 23220, USA

Email: B. Frank Gupton - bfgupton@vcu.edu

* Corresponding author

‡ These authors contributed equally to this article.

Additional experimental descriptions and NMR spectra

Table of Contents

General information……………………………………………………………………...……………………...S2

Synthesis of 5-iodopentan-2-one (**10**)…………………………….…………………...…...………………………S2

Synthesis of 5-(ethyl(2-hydroxyethyl)amino)pentan-2-one (**6**)……………...……………….....……………………...S3

Synthesis of 2-((4-((7-chloroquinolin-4-yl)amino)pentyl)(ethyl)amino)ethan-1-ol (**1**)…………….………………..S5

Spectra……………………………………………………………………………………..…………....S6–S10

General information

All reactions for the preparation of substrates performed in standard, dry glassware under an inert atmosphere of nitrogen or argon unless otherwise described. All starting materials and reagents were purchased from commercial sources and were used as received unless otherwise noted. ^{1}H and ^{13}C NMR spectra were recorded using 600 MHz spectrometers. Chemical shift (δ) values are given in ppm, and coupling constants (*J*) are given in Hz. The 7.26 ppm resonance of residual $CHCl_3$ (or 0 ppm of TMS) for proton spectra and the 77.23 ppm resonance of $CDCl_3$ for carbon spectra were used as internal references. Continuous-flow experiments were carried out using the E-series flow reactor instrument purchased from Vapourtec Ltd [1]. PFA tubing (1/16 OD x 1 mm ID) was used for all reactor coils in flow experiments. Most of the reagents and starting materials were purchased from commercial sources and used as received. All HPLC chromatograms were recorded on an Agilent Technologies 1260 Infinity instrument with a Poroshell 120 EC-C18 column (4.6 x 50 mm, 2.7 micron). Continuous flow hydrogenation was performed using a FlowCAT instrument [2].

Synthesis of 5-iodopentan-2-one (10):

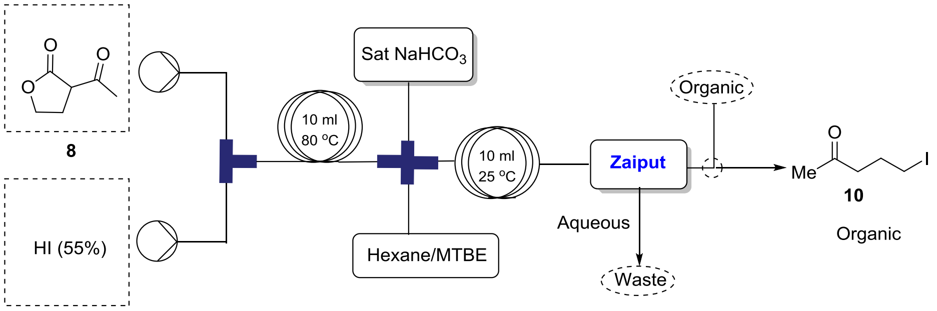

Two solutions, 2-acetylbutyrolactone **(1)** (1.176 mL, 10.35 mmol, 1.0 equiv) and Hydroiodic acid (55% aqueous sol) were pumped at 1.0 mL min^{-1} using peristaltic pumps through a 10 mL coil (residence time, $\mathbf{t}_R$ = 5 mins) at 80 °C. The crude mixture of **10** was streamed through a four valve mixer with sodium hydrogencarbonate at a flow rate of 1.0 mL min^{-1} and a mixture of hexanes/MTBE at 1.0 mL min^{-1}. The two phases were passed through a hydrophobic membrane-based separator (Zaiput, SEP-10) with dimensions 77 × 71 × 29 mm. The completion of the reaction was monitored using GC–MS. The organic phase was dried over anhydrous sodium sulfate and evaporated to dryness yielding the desired product as light brown liquid (14.72 g, 89%).

^{1}H NMR (600 MHz, $CDCl_3$): δ 3.22 (t, *J* = 6.9 Hz, 2H), 2.59 (t, *J* = 6.9 Hz, 2H), 2.17 (s, 3H), 2.06 (quin, *J* = 7.0 Hz, 2H); ^{13}C NMR (125 MHz, $CDCl_3$): δ 207.4, 44.0, 30.3, 27.2, 6.7; Spectra were obtained in accordance with those previously reported [3].

(Note: The Zaiput is made of 0.5 micron, polytetrafluoroethylene (PTFE) membrane filters. The membrane is stable for three runs, after, the KF is high. After three runs, the membrane filter needs to be changed).

Table S1: Optimization of 5-(ethyl(2-hydroxyethyl)amino)pentan-2-one in flow conditions (6)

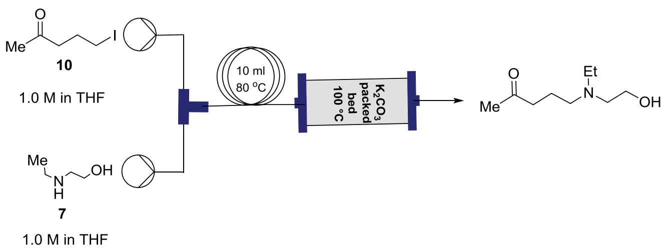

Entry	Concentration of **10 and 4**	Temperature (°C)	t_R =min	Conv[a] (%)
1	0.2 M	80	5	15%
2	0.2 M	80	10	18%
3	0.4 M	80	5	34
4	0.4 M	80	10	28
5	0.8 M	80	5	88
6	1.0 M	80	2.5	84
7	1.0 M	80	5	94 (86)[b]
8	1.0 M	80	10	86

[a] conversion determined by HPLC [b] Isolated yield

(Note: We used an OmniFit pack bed reactor having 100 × 10 mm ID, packed with K_2CO_3 (fine powder 325 mesh). The column was packed with a very large excess of base compared to total reagent processed. The base is consumed over a period of time. However, the first three runs do not affect the residence time. Our system was run for a total of three hours. More than 3 runs on 10.0 g scale reaction resulted in a change in the residence time and a stable back pressure of 4.0 bar.

Synthesis of 2-((4-aminopentyl)(ethyl)amino)ethan-1-ol (12) from 10:

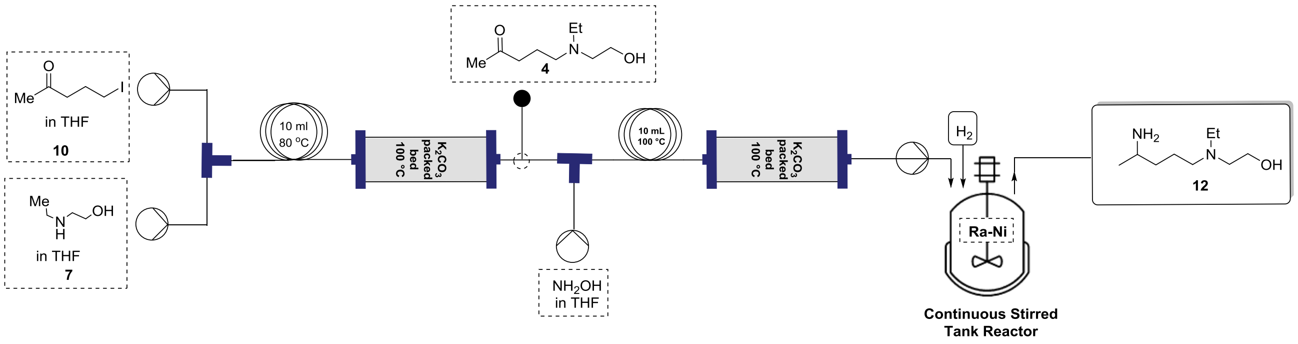

Telescope of compound 12 from 10: Prior to the start of the experiment, the flow reactor unit was rinsed with dry THF and flushed with nitrogen gas. At room temperature, the stock solutions of 5-iodopentan-2-one (**10**) (10.0, 1.0 M) and 2-(ethylamino)ethan-1-ol (**7**) in THF solution (1.0 M) were streamed in at 0.5 mL min^{-1} flow rates via a T-piece into a 10 mL reactor coil (t_R = 10 min) and passed through a packed bed reactor of potassium carbonate at 100 °C. The output solution was passed through a T-peace containing ammonium hydroxide solution were streamed 1.0 mL min^{-1}. via a T-piece into a 10 mL reactor coil (t_R = 10 min) and passed through a packed bed reactor of potassium carbonate at 100 °C. This solution was streamlined into a HEL continuous stirred tank reactor (CSTR) with a reaction volume of 150 mL. The reaction vessel was first charged with Raney-Nickel (1.0g). The reaction pressure was set to 10 bar of hydrogen pressure supplied by hydrogen gas (ultrahigh purity) at a flow rate of 0.5 mL min^{-1}. The reaction temperature was set to 80 °C, which was controlled by a thermocouple positioned in the reaction mixture. The reaction was stirred at 750 rpm mechanical stirring to provide proper mixing. Two thermocouples were used to control the reaction volume in the reactor by setting a level control of −3 °C. The lower thermocouple constantly measured and controlled the reaction temperature and the upper thermocouple measured the temperature at approximately 150 mL reactor volume. When the two thermocouples were within 3 °C the level control 'opened' the exit stream dip tube to allow products to exit the reactor or 'closed' the exit stream dip tube to allow the reactor to fill when the temperature difference between the two thermocouples was greater than 3 °C. The product was collected after a full reaction volume of material (150 mL) had passed through the CSTR indicating that steady-state was reached. The reaction was monitored by liquid chromatography and ^{1}H NMR spectra. The reaction mixture was filtered through a celite pad and dried the reaction mixture. The solution was extracted with water (10 mL) and dichloromethane (3 × 20 mL). The organic layers were combined, washed with brine and dried over sodium sulfate and evaporated in vacuo and did fractional distillation to give a colorless liquid (9.29 g, 68%).
(Note: hydroxylamine hydrochloride was free based by treating with potassium carbonate)

Table S2: Solvent and base screening synthesis of 2-((4-((7-chloroquinolin-4-yl)amino)pentyl)(ethyl)amino)ethan-1-ol (1):

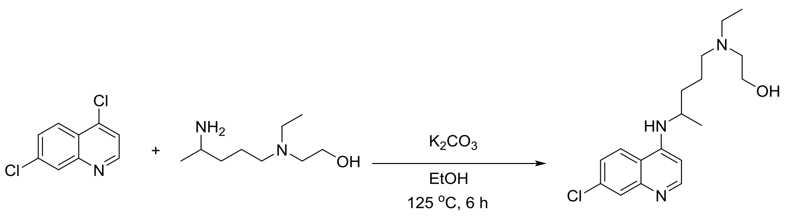

**Base**	Temperature (°C)	**Solvent**	**Conv (%)[a]**
K_2CO_3	125	IPA	34
K_2CO_3	125	MeOH	64
K_2CO_3	125	EtOH	82
K_2CO_3	125	DMF	0
K_2CO_3	125	THF	50
NaH	125	Dioxane	20
LiHMDS	125	Dioxane	15
NaOH	125	Dioxane	34

[a] conversion determined by HPLC and ^{1}H NMR

^{1}H NMR and ^{13}C NMR spectra:

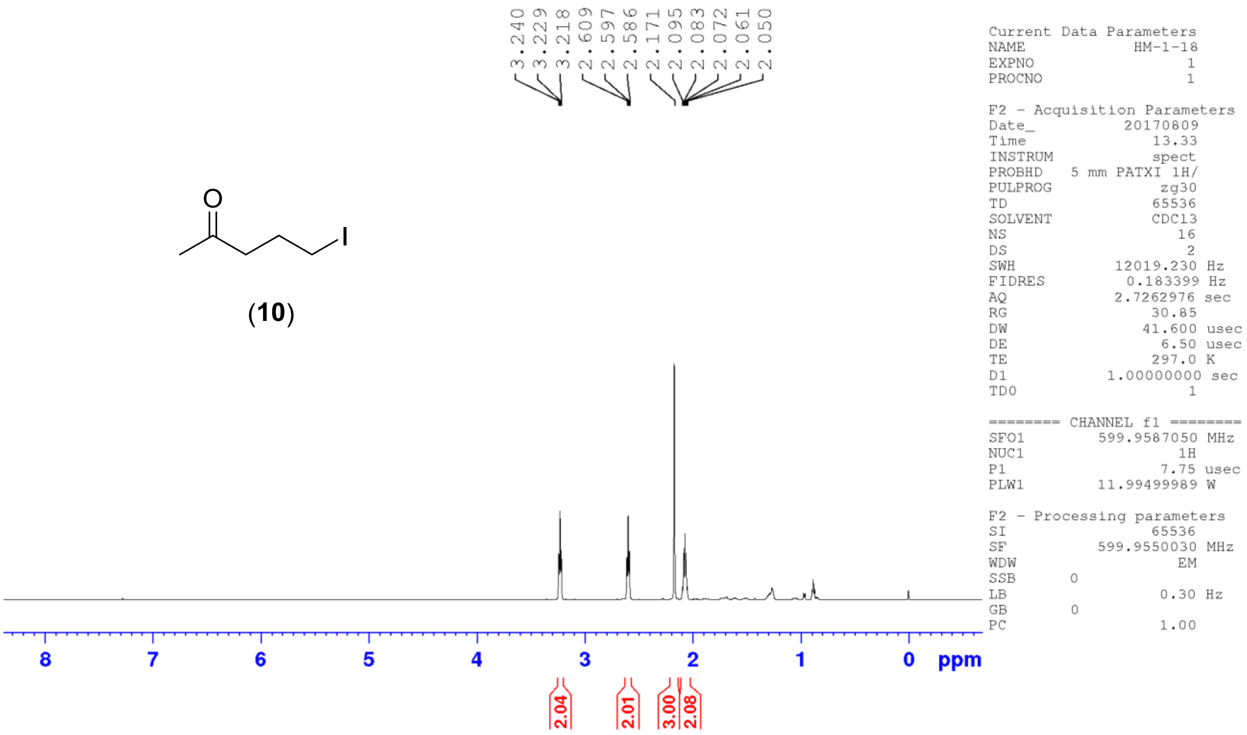

^{1}H NMR Spectra of compound **10**

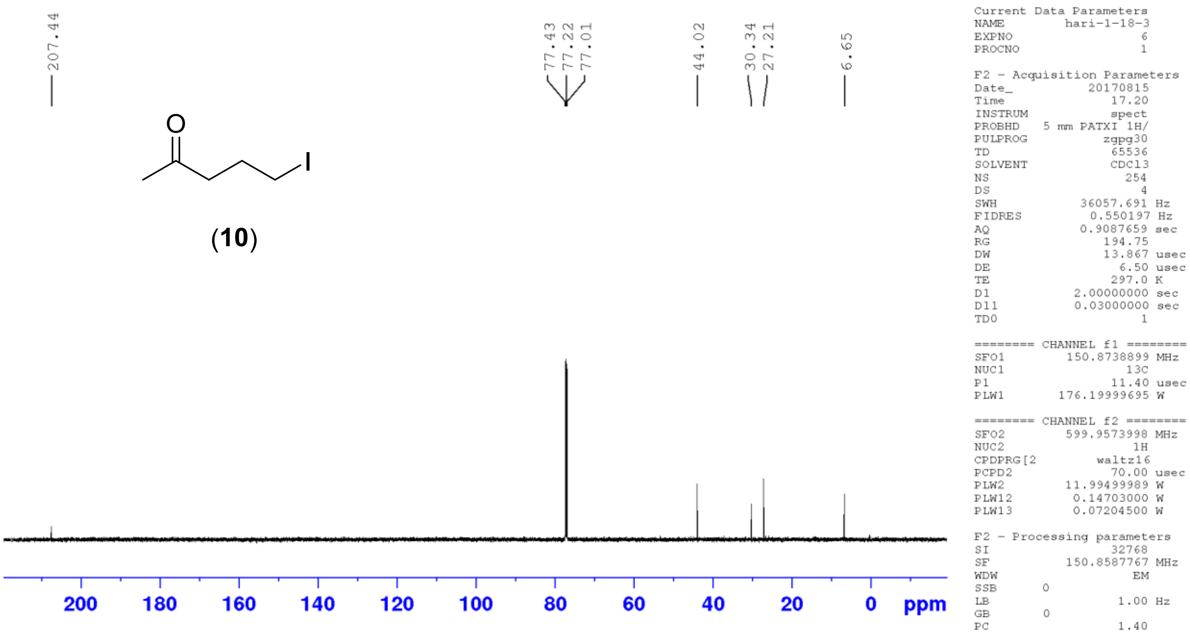

^{13}C NMR Spectra of compound **10**

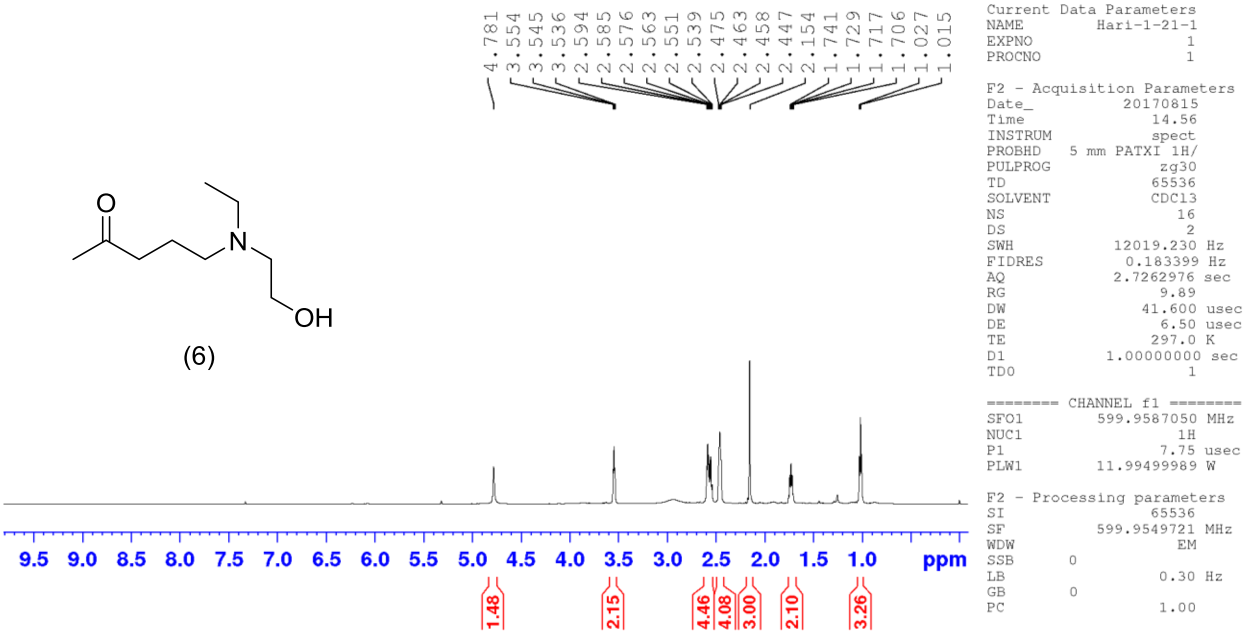

^{1}H NMR Spectra of compound **6**

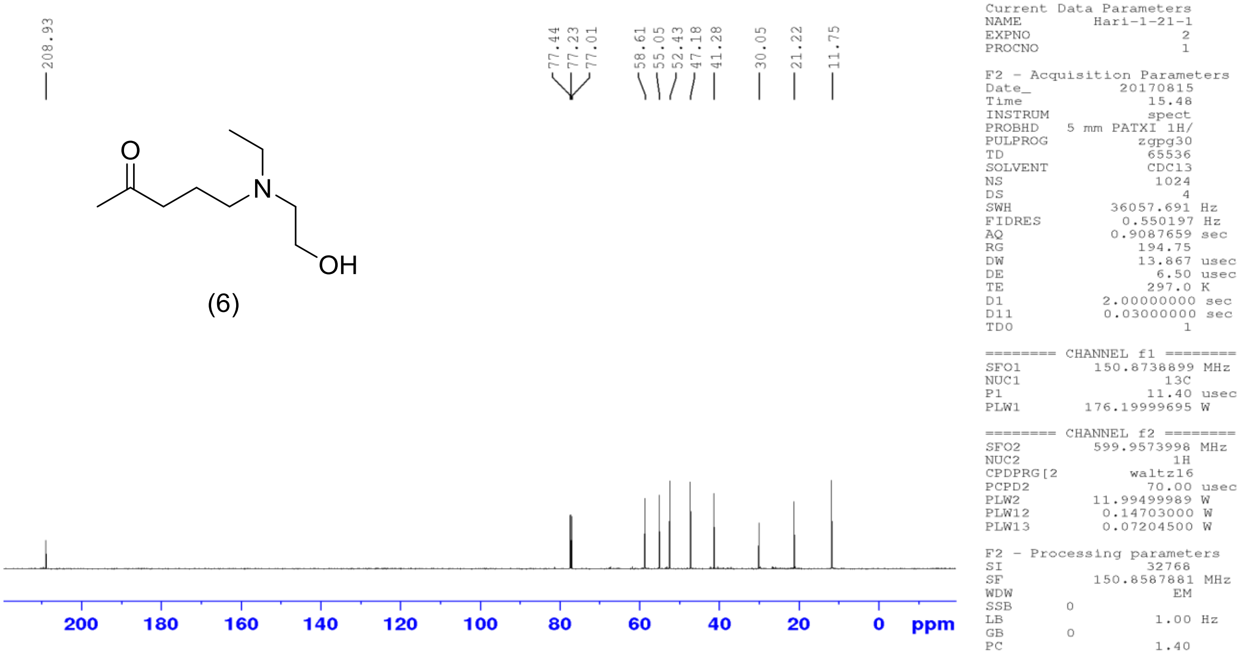

^{13}C NMR Spectra of compound **6**

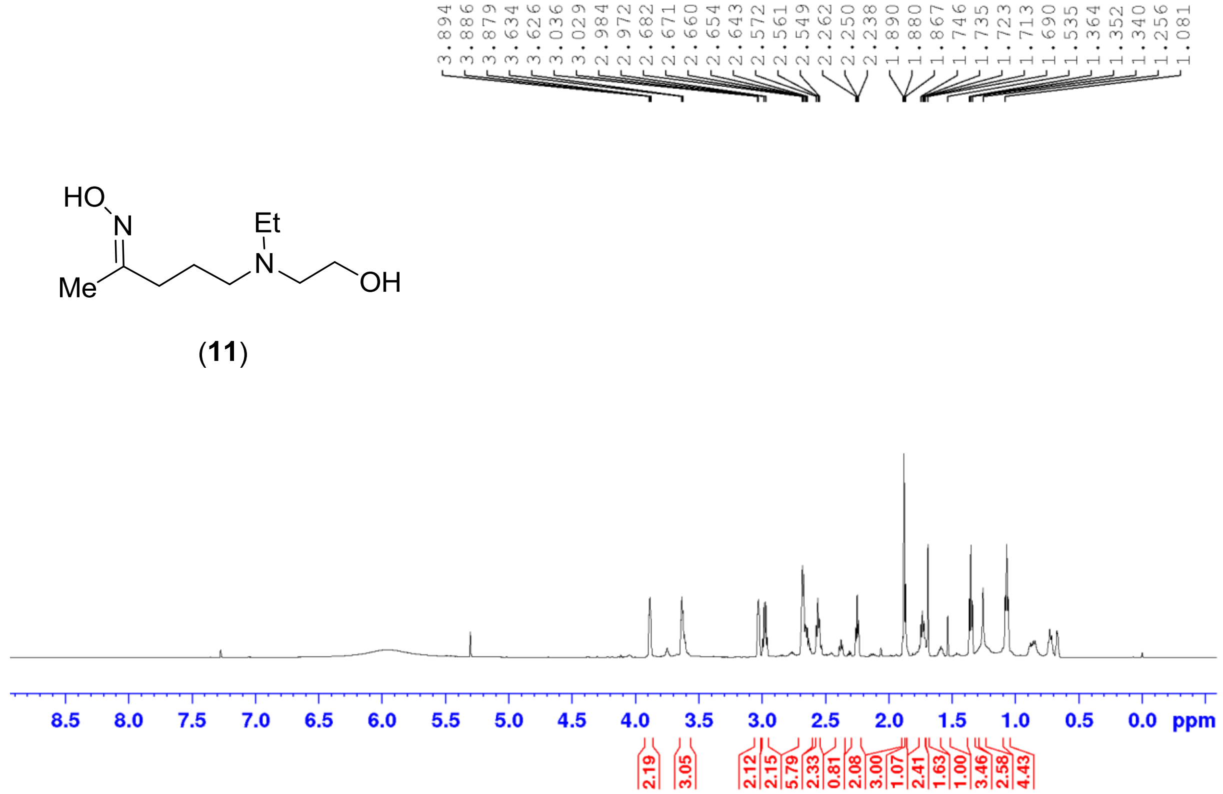

^{1}H NMR Spectra of compound **11**

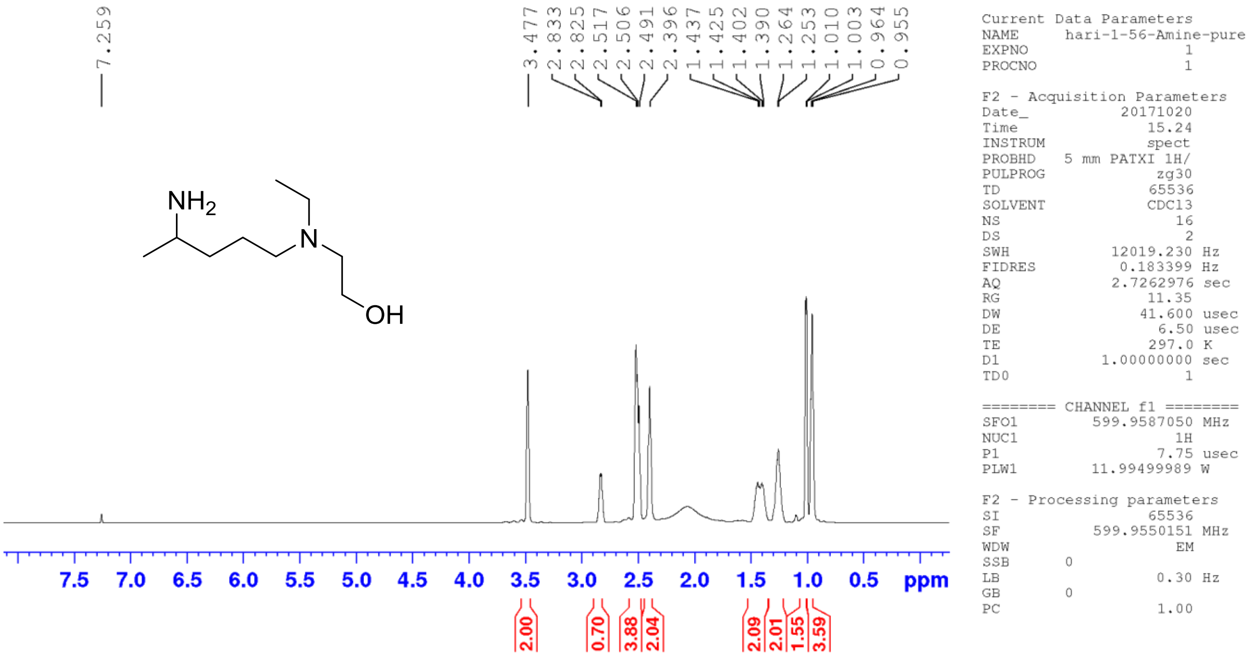

^{1}H NMR Spectra of compound **12**

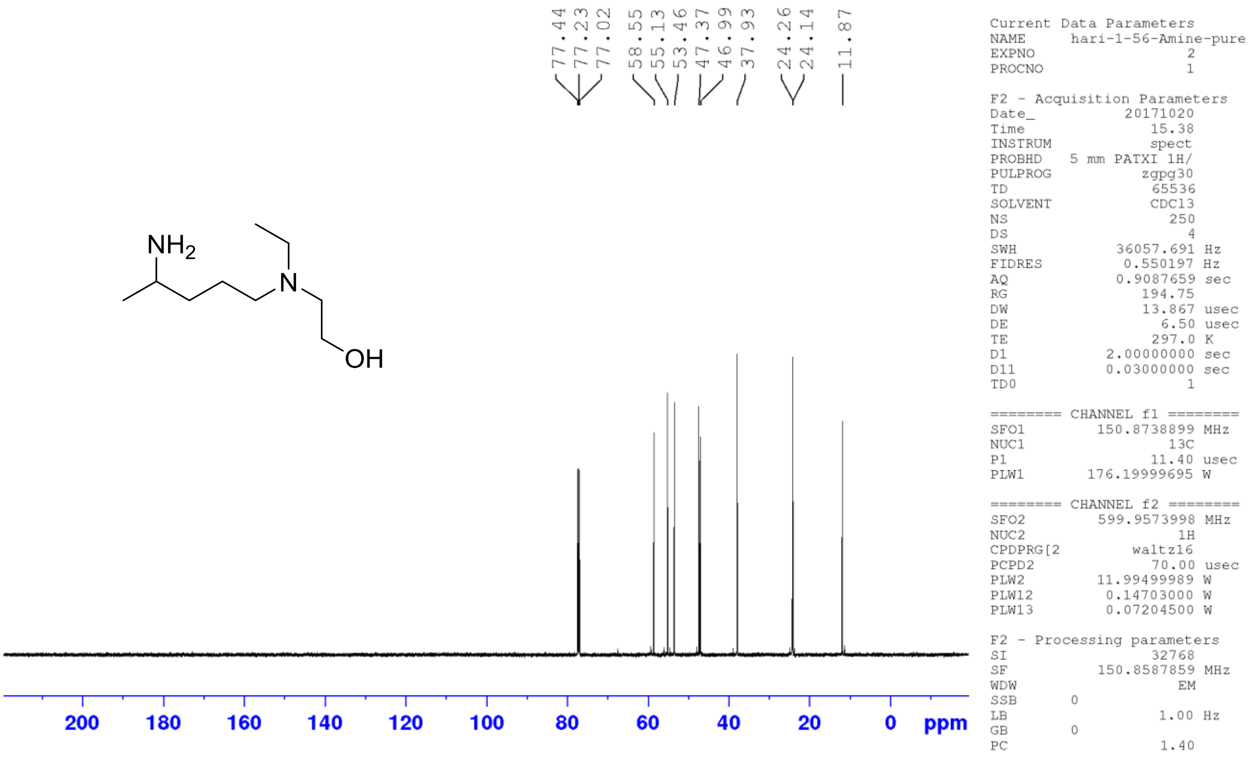

^{13}C NMR Spectra of compound **12**

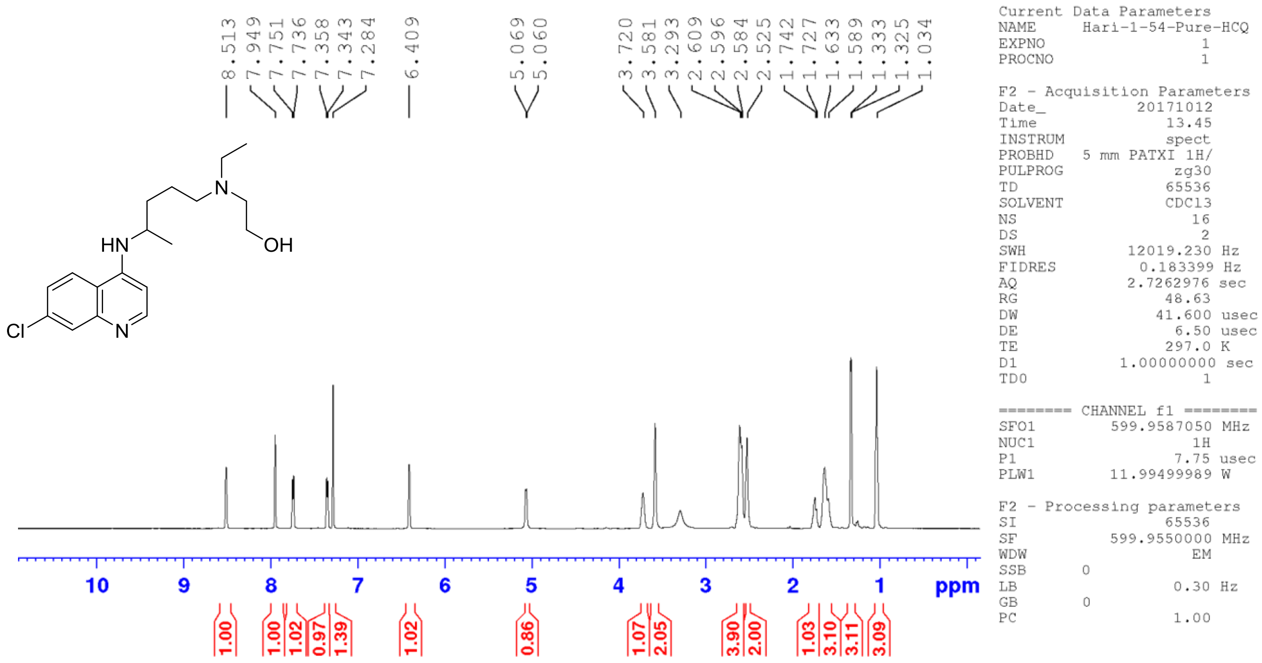

^{1}H NMR Spectra of compound **1**

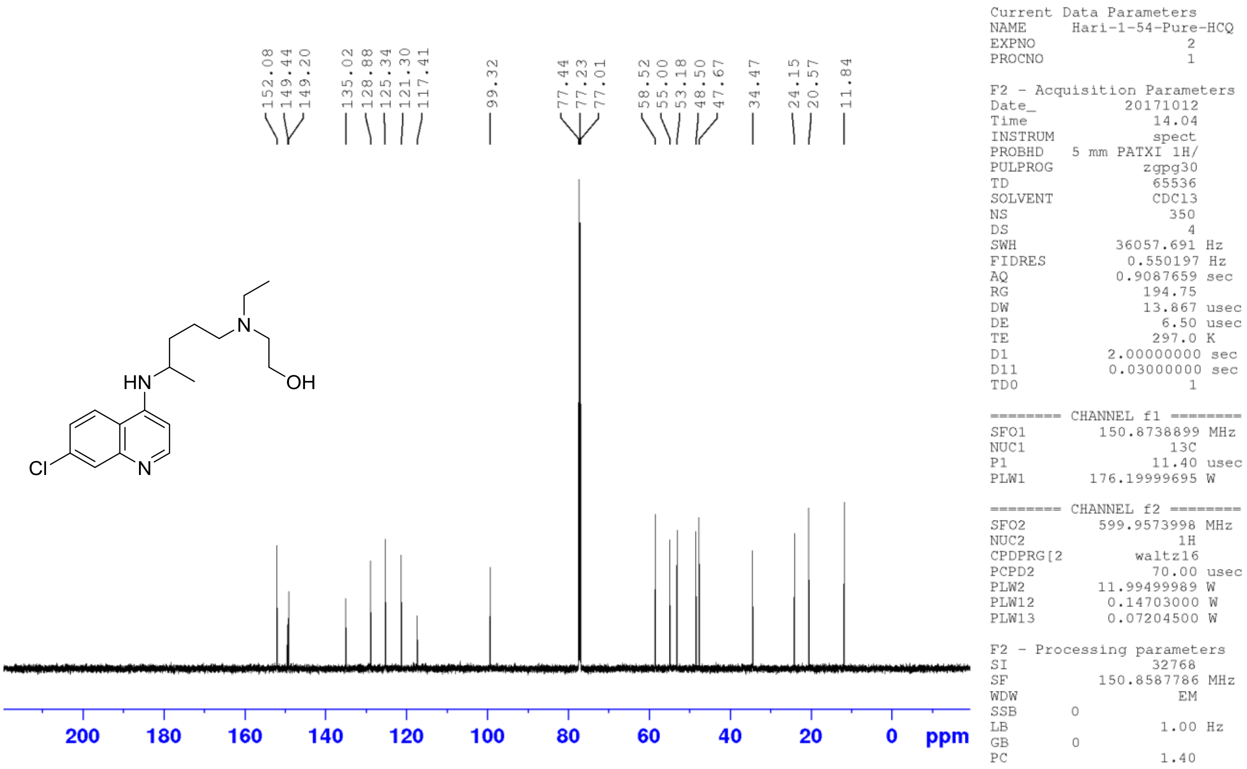

^{13}C NMR Spectra of compound **1**

References

1. For more information on the Vapourtec E-series please visit: https://www.vapourtec.com/.
2. Continuous flow chemistry catalytic reactions with FlowCAT: http://www.helgroup.com/reactor-systems/hydrogenation-catalysis/flowcat/
3. a) Cornish, C. A.; Warren, S.; *J. Chem. Soc. Perkin Trans. I. Organic and Bio-Organic Chemistry*. **1985**, *12*, 2585-2598. doi:10.1039/P19850002585. b) Muntedt, R.; Wannagat, U.; Wrobel, D. *J. Organomet. Chem.* **1984**, *264*, 135-148. doi.org/10.1016/0022-328X(84)85139-6
4. a) Glenn, J. S.; Pham, E. A. PCT Int. Appl. WO2017004454 A1, 2017, 55pp. b) You, H.; Liu, Yu; Ning, Fan; Zheng, Zhuo; Yu, Qiaolu; Niu, Xiaobin; Li, Cheng. 2015, CN104803859A, 6pp. c) Min, Y. S.; Cho, Hyun-Sung; Mo, K. W. PCT Int. Appl.,WO 2010027150 A2, 2010, 9pp d) Blaney, P. M.; Byard, S. J.; Carr, G.; Ellames, G. J.; Herbert, J. M.; Peace, J. E.; Smith, D. I. *Tetrahedron: Asymmetry*. 1994, 5, 1815-1822. doi: 10.1016/0957-4166(94)80090-1.
